# Mitochondrial Genetics and Epigenetics in Osteoarthritis

**DOI:** 10.3389/fgene.2019.01335

**Published:** 2020-01-17

**Authors:** Ignacio Rego-Pérez, Alejandro Durán-Sotuela, Paula Ramos-Louro, Francisco J. Blanco

**Affiliations:** Grupo de Investigación en Reumatología. Instituto de Investigación Biomédica de A Coruña (INIBIC), Complexo Hospitalario Universitario de A Coruña (CHUAC), Sergas, Universidade da Coruña (UDC), A Coruña, Spain

**Keywords:** mitochondria, genetics, epigenetics, osteoarthritis, methylation

## Abstract

During recent years, the significant influence of mitochondria on osteoarthritis (OA), the most common joint disease, has been consistently demonstrated. Not only mitochondrial dysfunction but also mitochondrial genetic polymorphisms, specifically the mitochondrial DNA haplogroups, have been shown to have an important influence on different OA-related features, including the prevalence, severity, incidence, and progression of the disease. This influence could probably be mediated by the role of mitochondria in the regulation of different processes involved in the pathogenesis of OA, such as energy production, the generation of reactive oxygen and nitrogen species, apoptosis, and inflammation. The regulation of these processes is at least partially controlled by the bi-directional communication between the nucleus and mitochondria, which permits the regulation of adaptation to a wide range of stressors and the maintenance of cellular homeostasis. This bi-directional communication consists of an “anterograde regulation” by which the nucleus regulates mitochondrial biogenesis and activity and a “retrograde regulation” by which both mitochondria and mitochondrial genetic variation exert a regulatory signaling control over the nuclear epigenome, which leads to the modulation of nuclear genes. Throughout this mini review, we will describe the evidence that demonstrates the profound influence of the mitochondrial genetic background in the pathogenesis of OA, as well as its influence on the nuclear DNA methylome of the only cell type present in the articular cartilage, the chondrocyte. This evidence leads to serious consideration of the mitochondrion as an important therapeutic target in OA.

## Introduction

Osteoarthritis (OA) is the most common chronic progressive disorder that involves movable joints, occurring in 10-20% of the population over 50 years of age; the incidence of OA is estimated to double within the next 30 years (Blanco et al., 2011). The pathogenesis of OA is characterized by extracellular matrix degradation and cell stress initiated by micro- and macro-injuries that lead to the activation of maladaptive repair responses, including proinflammatory pathways of innate immunity. The disease manifests first as a molecular derangement (abnormal tissue metabolism) followed by anatomical and/or physiological derangements (characterized by cartilage degradation, bone remodeling, osteophyte formation, joint inflammation, and the loss of normal joint function) that can culminate in illness (Kraus et al., 2015). OA is actually considered a disease of the whole joint as an organ, resulting in organ dysfunction or joint failure (Blanco, 2014). In addition, this disease is one of the most common reasons for visits to primary care physicians and is also the leading cause of permanent work incapacitation. OA has no effective treatment today, and joint replacement is the only choice in cases of total joint dysfunction.

The nature of OA is heterogeneous, because a combination of factors such as occupation, age, gender, body mass index, and genetics have a profound influence on its pathogenesis. As a consequence of this, several different phenotypes characterize this disease, including inflammatory, aging-related, metabolic, pain, and post-traumatic phenotypes (Berenbaum, 2013; Sellam and Berenbaum, 2013; Blanco et al., 2018). In agreement with this heterogeneous nature, it has also been proposed that, on the one hand, mitochondria and mitochondrial DNA (mtDNA) have an important impact on the development of OA (Valdes and Goldring, 2017; Blanco et al., 2018; Li et al., 2019) and, on the other hand, that epigenetics is one of the main actors involved in the phenotypic modulation that articular chondrocytes, the only cell type present in articular cartilage, undergo during the OA process (Roach et al., 2005; Reynard and Loughlin, 2012; Blanco and Rego-Pérez, 2014). In this sense, it has also been consistently shown that specific mtDNA polymorphisms, called mtDNA haplogroups, have been associated not only with different OA-related features such as the incidence or progression of the disease (Fernandez-Moreno et al., 2017a; Fernandez-Moreno et al., 2017b; Koo et al., 2019) but also with the differential methylation status of articular cartilage (Cortes-Pereira et al., 2019). In light of the involvement of mitochondrial DNA in cell behavior and metabolism (Wallace et al., 1999; Martínez-Redondo et al., 2010), in this review, we discuss some of the evidence implicating the epigenetic effect of mtDNA variation in the pathogenesis of OA.

## Mitochondria in Osteoarthritis

Because articular chondrocytes are highly glycolytic cells that obtain their energy mainly from anaerobic glucose metabolism, the role of mitochondria in the pathogenesis of OA was not studied in depth until early 2000. However, a large number of studies have demonstrated the profound influence of mitochondria in the pathogenesis of OA.

Terkeltaub and co-workers published a full review describing how mitochondrial impairment of chondrocytes is an important mediator of the establishment of OA (Terkeltaub et al., 2002). Specifically, these and other authors described that mitochondrial dysfunction mediates several specific pathogenic pathways implicated in the OA process, including oxidative stress, chondrocyte apoptosis, cartilage matrix calcification, autophagy, impaired anabolic and growth response of chondrocytes, and increased cytokine-induced inflammation (Terkeltaub et al., 2002; Blanco et al., 2004; Lotz and Loeser, 2012; López de Figueroa et al., 2015). In agreement with these findings, our group also demonstrated that, compared with healthy chondrocytes from patients of the same age, osteoarthritic chondrocytes have reduced mitochondrial activity, mainly in complexes II and III, and increased mitochondrial mass (Maneiro et al., 2003). In addition, the apoptotic mitochondrial pathway is implicated in the apoptosis of osteoarthritic chondrocytes (Hwang and Kim, 2015), and the inhibition of mitochondrial complexes III and V increases the mitochondrial-mediated inflammatory response in OA chondrocytes mediated by an overproduction of reactive oxygen species (ROS) (Vaamonde-García et al., 2012). Mitochondrial dysfunction has also been associated with a significant downregulation of superoxide dismutase 2 (SOD2) (Gavriilidis et al., 2013), one of the major mitochondrial antioxidant proteins, whose levels are also diminished in the superficial layers of end-stage OA cartilage (Ruiz-Romero et al., 2009; Scott et al., 2010).

Mitophagy is a form of autophagy, a process that involves the removal of damaged macromolecules and organelles to regulate cell homeostasis (Choi et al., 2013); specifically, mitophagy consists of the elimination of depolarized and damaged mitochondria. Different studies have demonstrated that the activation of this process protects against mitochondrial dysfunction, prevents ROS production and improves chondrocyte survival under pathological conditions (López de Figueroa et al., 2015; Ansari et al., 2018). It has also been demonstrated that mitochondrial biogenesis is deficient in human OA chondrocytes, leading the chondrocyte to adopt procatabolic responses; however, the activation of the AMP-activated protein kinase (AMPK)-NAD-dependent protein deacetylase sirtuin-1 (SIRT1)-peroxisome proliferator-activated receptor γ co-activator 1α (PGC1α) pathway reverses impaired mitochondrial biogenesis, which is mediated by mitochondrial transcriptional factor A (TFAM) (Wang et al., 2015).

## mtDNA Haplogroups and Osteoarthritis

Mitochondria are considered unique organelles because they contain their own maternally inherited DNA, the mtDNA, containing 2 rRNAs, 22 tRNAs, and 13 essential mitochondrial protein-coding genes. The mutation rate of this circular molecule is higher than that of the nuclear DNA, mainly due to i) its proximity to the main source of ROS production, ii) the lack of an efficient repair system, and iii) the higher replication rate of mtDNA (Li et al., 2019). As a consequence of this, mtDNA sequences evolved by sequentially accumulating functional mutations along radiating maternal lineages when humans migrated out of Africa and adapted their energy metabolism to different environments, giving rise to mtDNA haplogroups (Torroni et al., 1996; Wallace, 2016). However, these types of mtDNA variants, though providing an important degree of adaptation as people migrated into new environments, might also have contributed to modern human disorders such as hypertension, diabetes, obesity, or neurodegenerative diseases (Ruiz-Pesini et al., 2004; Marom et al., 2017).

In this sense, OA is not an exception. During recent years, different studies have shown clinical associations between specific mtDNA haplogroups and different OA-related features, including the prevalence, progression, and incidence of the disease (**Table 1**).

**Table 1:** Published associations of mtDNA variants with specific OA-related features.

Study cohort	Population	Haplogroup	OR (95%CI) p-value/effect on the biomarker	Reference
**OA prevalence**				
Spanish	457 OA cases, 262 controls	J	OR = 0.460 (0.282-0.748) p = 0.002	(Rego-Perez et al., 2008)
		JT	OR = 0.564 (0.384-0.828) p = 0.005	
Spanish	550 OA cases, 505 controls	J	OR = 0.519 (0.271-0.994) p = 0.048	(Rego et al., 2010)
UK	453 OA cases, 280 controls	T	OR = 0.574 (0.350-0.939) p = 0.027	(Soto-Hermida et al., 2014a)
UK	7846 OA cases, 5402 controls	J	OR = 1.190 (0.720-1.950) ns ^&^	(Hudson et al., 2013)
Meta-analysis	2557 OA cases, 1339 controls	J	OR = 0.570 (0.460-0.710) p < 0.0001	(Shen et al., 2014)
	2478 OA cases, 1173 controls	JT	OR = 0.700 (0.580-0.840) p = 0.0002	
Chinese	187 OA cases, 420 controls	G	OR = 3.834 (1.139-12.908) p = 0.003	(Fang et al., 2014)
		B	OR = 0.503 (0.283-0.893) p = 0.019	
**OA progression**				
OAI	891 knee OA cases	T	HR = 0.499 (0.261-0.819) p < 0.05	(Soto-Hermida et al., 2014b)
Spanish	281 knee OA cases	JT*	HR = 0.584 (0.354-0.964) p = 0.036	(Soto-Hermida et al., 2015)
CHECK	431 knee OA cases	T	HR = 0.645 (0.419-0.978) p < 0.05	(Fernandez-Moreno et al., 2017b)
		JT	HR = 0.707 (0.501-0.965) p < 0.05	
Meta-analysis	1603 knee OA cases	T	HR = 0.612 (0.454-0.824) p = 0.001	(Fernandez-Moreno et al., 2017b)
		JT	HR = 0.765 (0.624-0.938) p = 0.009	
**OA incidence**				
OAI	2579 subjects	J	HR = 0.680 (0.470-0.968) p < 0.05	(Fernandez-Moreno et al., 2017a)
CHECK	635 subjects	J	HR = 0.728 (0.469-0.998) p < 0.05	(Fernandez-Moreno et al., 2017a)
Meta-analysis	3214 subjects	J	HR = 0.702 (0.541-0.912) p = 0.008	(Fernandez-Moreno et al., 2017a)
Korean	438 subjects	B	RR = 2.389 (1.315-4.342) *p =* 0.004	(Koo et al., 2019)
**OA biomarkers**				
Spanish	73 knee OA cases, 77 controls	J	Decreased serum levels of catabolic type II collagen biomarkers^&^	(Rego-Perez et al., 2010)
		H	Increased serum levels of catabolic type II collagen biomarkers^&^	
Spanish	73 knee OA cases, 77 controls	J	Decreased serum levels of MMP-13^&^	(Rego-Perez et al., 2011)
		H	Increased serum levels of MMP-13 and MMP-3^&^	
Spanish	79 knee OA cases, 166 controls	J	Lower NO production	(Fernandez-Moreno et al., 2011)
OAI	255 knee OA cases	J	Fewer large tibiofemoral BMLs	(Rego-Perez et al., 2018)

mtDNA, mitochondrial DNA; UK, United Kingdom; OA, Osteoarthritis; OAI, Osteoarthritis Initiative; CHECK, Cohort Hip and Cohort Knee; NO, nitric oxide; BMLs, bone marrow lesions; MMP, metalloproteinase; OR, odds ratio; HR, hazard ratio; RR, risk ratio; ns, non-significant; (*) when compared with mtDNA cluster KU; ^(&)^ p-value after multiple testing correction.

### mtDNA Haplogroups and the Prevalence of OA

Different studies have shown significant associations between specific mtDNA haplogroups and the prevalence of OA. Specifically, mtDNA variants belonging to the European cluster JT have been associated with a lower risk of knee and hip OA in a cohort of Spanish patients (Rego-Perez et al., 2008; Rego et al., 2010). mtDNA haplogroup T was also associated with a decreased risk of knee OA in a population from the United Kingdom (Soto-Hermida et al., 2014a); however, a later study in a larger population cohort from the same country failed to replicate these findings (Hudson et al., 2013). In addition, Asian mtDNA haplogroups B and G have been described as protective and risk factors respectively for knee OA in a population of southern China (Fang et al., 2014). The authors have proposed that the mechanisms that could explain that association are related to the alteration of both mitochondrial function and OA-related signaling pathways (Fang et al., 2016). A meta-analysis summarizing most of the studies described above concluded that mtDNA cluster JT is associated with a lower risk of OA prevalence in Spanish populations (Shen et al., 2014).

A plausible cause for the lack of replication among different case-control studies, even when nuclear DNA polymorphisms are analyzed, could be the unavailability of knee (or hip) radiographs for most population-based controls. This is not a minor issue, since up to 50% of people without joint symptoms may develop radiographic changes comparable to OA (Hannan et al., 2000). In this sense, our group has always considered the radiological status, because we believe that both mitochondrial dysfunction and mtDNA variation have a greater impact on the evolution of joint structure than on pain.

### mtDNA Haplogroups and Radiographic OA Progression and Incidence

The use of well-characterized prospective cohorts of patients permits rigorous studies to analyze the influence of mtDNA haplogroups on the rate of progression and incidence of OA over time. mtDNA variants within mitochondrial cluster JT were associated with lower rates of radiographic knee OA progression in different world populations, including Spain, the USA, and the Netherlands. Specifically, in a Spanish cohort, mtDNA cluster JT was associated with a lower rate of radiographic progression in terms of Kellgren and Lawrence grade (Kellgren and Lawrence, 1957), and even patients with haplogroup H were more prone to requiring total joint replacement (Soto-Hermida et al., 2015). mtDNA haplogroup T was associated with a decreased rate of radiographic knee OA progression, as well as a reduced loss of knee cartilage integrity over time in patients of the Osteoarthritis Initiative (OAI) of the US National Institutes of Health (NIH) (Soto-Hermida et al., 2014b). This association was then replicated in the CHECK cohort (cohort hip and cohort knee), another prospective cohort of OA patients from the Netherlands (Fernandez-Moreno et al., 2017b). Finally, a subsequent meta-analysis including the above-mentioned studies confirmed that mtDNA variants of the JT cluster act as protective factors against the radiographic progression of the disease (Fernandez-Moreno et al., 2017b).

In terms of disease incidence, a meta-analysis including 3217 individuals from the OAI and CHECK cohorts concluded that, compared with the most common Caucasian haplogroup H, subjects with mtDNA haplogroup J show a lower rate of incident knee OA over an eight-year period (Fernandez-Moreno et al., 2017a). This study included the design of a cellular model of transmitochondrial cybrids, consisting of cells with a defined and uniform nuclear background containing mitochondria from different sources, to demonstrate the existence of functional differences between haplogroups H and J; the study concluded that, compared with H cybrids, J cybrids produce less ATP, but this was accompanied with lower amounts of peroxynitrite and mitochondrial superoxide anion together with a lower rate of apoptosis under stress conditions as well as an increased ability to cope with oxidative stress (Fernandez-Moreno et al., 2017a). Another study in Korean populations showed that Asian mtDNA haplogroup B, described as a protective factor against knee OA prevalence in a population from the south of China (Fang et al., 2014), was a risk factor for the incidence of knee OA over an eight-year period (Koo et al., 2019).

### mtDNA Haplogroups and Biomarkers in OA

In an effort to detect structural changes in an early stage of the disease, to monitor disease progression, or even to assess therapeutic responses with more sensitivity and reliability, molecular biomarkers have been developed in OA (Garnero et al., 2002; Rousseau and Delmas, 2007; Camacho-Encina et al., 2019). In a set of clinically relevant studies, OA-protective haplogroup J has been significantly associated with lower serum levels of catabolic type II collagen biomarkers and matrix metalloproteinases, in contrast to haplogroup H carriers, which showed significantly higher levels (Rego-Perez et al., 2010; Rego-Perez et al., 2011). Despite not being considered a biomarker of the disease, although higher-than-normal production has been described in OA chondrocytes (Maneiro et al., 2005; Henrotin and Kurz, 2007), the production of nitric oxide (NO) is significantly lower in articular chondrocytes harboring mtDNA haplogroup J than in non-J chondrocytes (Fernandez-Moreno et al., 2011). Based on these findings, haplogroups J and H represent two different OA phenotypes, leading to the consideration of these mtDNA haplogroups as complementary genetic biomarkers of the disease (Fernandez-Moreno et al., 2012).

In terms of imaging biomarkers, the identification and quantification of early bone marrow lesions (BMLs) has great relevance for assessing symptomatic progression and radiographic worsening over time in patients with OA (Roemer et al., 2016). In this sense, a longitudinal study including 255 participants from the OAI cohort that developed incident knee OA at 48 months revealed that patients with mtDNA haplogroup J were less likely to develop large BMLs in the tibiofemoral compartment of the knee than those with mtDNA haplogroup H (Rego-Perez et al., 2018). This association could be due to the differential behavior of mtDNA haplogroups H and J in terms of the metabolic activity and inflammation that takes place in BMLs (Kuttapitiya et al., 2017).

In summary, in Caucasian populations, it seems that haplogroups belonging to mitochondrial cluster JT are protective against OA and have even been associated with increased longevity in some European populations (Dato et al., 2004). However, in energy-deficiency diseases, such as LHON (Leber Hereditary Optic Neuropathy) these haplogroups, specifically haplogroup J, are risk factors (Hudson et al., 2007). This controversy is potentially related to the uncoupling nature of the genetic polymorphisms associated with these haplogroups, by which ATP production is reduced but, conversely, lower ROS generation, oxidative damage, and apoptosis are also expected (Ruiz-Pesini et al., 2004).

## The Mitochondrial Genome as an Epigenetic Regulator in Articular Cartilage

It is well known that bi-directional communication exists between the nucleus and mitochondria with the aim of maintaining cellular homeostasis and regulating adaptation to a broad range of stressors (Quirós et al., 2016; Wallace, 2016). This communication implies, on the one hand, that mitochondria are controlled by the nucleus by means of an “anterograde regulation,” a mechanism that regulates mitochondrial activity and biogenesis to provide cellular needs; on the other hand, mitochondria and mtDNA variation maintain partial regulatory signaling control over the nucleus through a “retrograde regulation,” which leads to the modification of cellular metabolism and function by activating the expression of nuclear genes with the aim of protecting against mitochondrial dysfunction (Jazwinski, 2013; Horan and Cooper, 2014; Matilainen et al., 2017).

Based on the above findings, and taking into account the reported associations between common mtDNA variants and different physiological and pathological phenotypes (Gómez-Durán et al., 2010; Martínez-Redondo et al., 2010; Marom et al., 2017), it can be deduced that interactions between mtDNA sequences, nuclear DNA, and the environment have important effects on mammalian biology (Kenney et al., 2014). In this sense, the effect of specific nuclear polymorphisms classically identified as risk factors for different diseases, such as Parkinson´s disease, cancer, and severe cardiopathy, is also modulated by mtDNA variation (Maruszak et al., 2008; Strauss et al., 2013; Blein et al., 2015). In the case of OA, as subjects carrying mtDNA haplogroup J are not fully protected from suffering from the disease, it would be interesting to investigate potential interactions between this haplogroup, as well as the biochemically opposite haplogroup H (Wallace et al., 1999; Martínez-Redondo et al., 2010), and the most robust nuclear polymorphisms described in different GWAS performed in OA (Warner and Valdes, 2017; Zengini et al., 2018; Tachmazidou et al., 2019).

In terms of animal models, interesting work in conplastic mice (mice with a constant nuclear background but different mtDNA genomes) showed profound differences in health longevity between conplastic strains. The level of divergence between the two strains, equivalent to that between human African and Eurasian mtDNAs, showed different behavior in terms of mitochondrial proteostasis, reactive oxygen generation, obesity, and insulin signaling as well as in cell-senescence-related parameters such as telomere shortening and mitochondrial dysfunction (Latorre-Pellicer et al., 2016). Most of the altered processes described in the work of Latorre-Pellicer and co-workers are also involved in many common human diseases. In agreement with this, preliminary analyses of OA-related features using the two strains of these animals revealed significant differences between them in terms of the expression of the autophagy-related protein microtubule-associated protein 1 light chain 3 (LC3) and extracellular matrix-degrading protein metalloproteinase-13 (MMP-13) as well as significant differences in the Mankin score, a scoring system for the histopathological classification of the severity of cartilage lesions in OA (Scotece et al., 2019).

In this context of mitochondrial-nuclear interactions, epigenetics emerges as an important mechanism. Specifically, DNA methylation is the best-characterized epigenetic mechanism in OA. DNA methylation consists of the addition of a methyl group (CH_3_) by S-adenosyl-methionine (SAM) to a cytosine that lies at 5´ of guanine (CpG site) to give rise to methylated cytosine. When this process occurs in high-density CpG regions of promoters, gene silencing occurs; in contrast, when methylation occurs in gene bodies, it leads to increased gene expression (Hellman and Chess, 2007). In contrast to nuclear DNA, the effects of mtDNA methylation in OA have not been explored so far; however, it is well accepted that mtDNA variation is able to modulate the nuclear methylome. Consistent with this concept, two independent studies using transmitochondrial cybrids showed that global DNA methylation levels are differentially modulated by mtDNA haplogroups J and H (Bellizzi et al., 2012; Atilano et al., 2015); in addition, these two haplogroups also mediate the methylation profile and the expression levels of genes involved in angiogenesis, inflammation, and other signaling pathways (Atilano et al., 2015). Moreover, different haplogroups on a uniform nuclear background of mouse embryonic stem cells were also associated with different methylation profiles and gene expression (Kelly et al., 2013).

**Figure 1:**
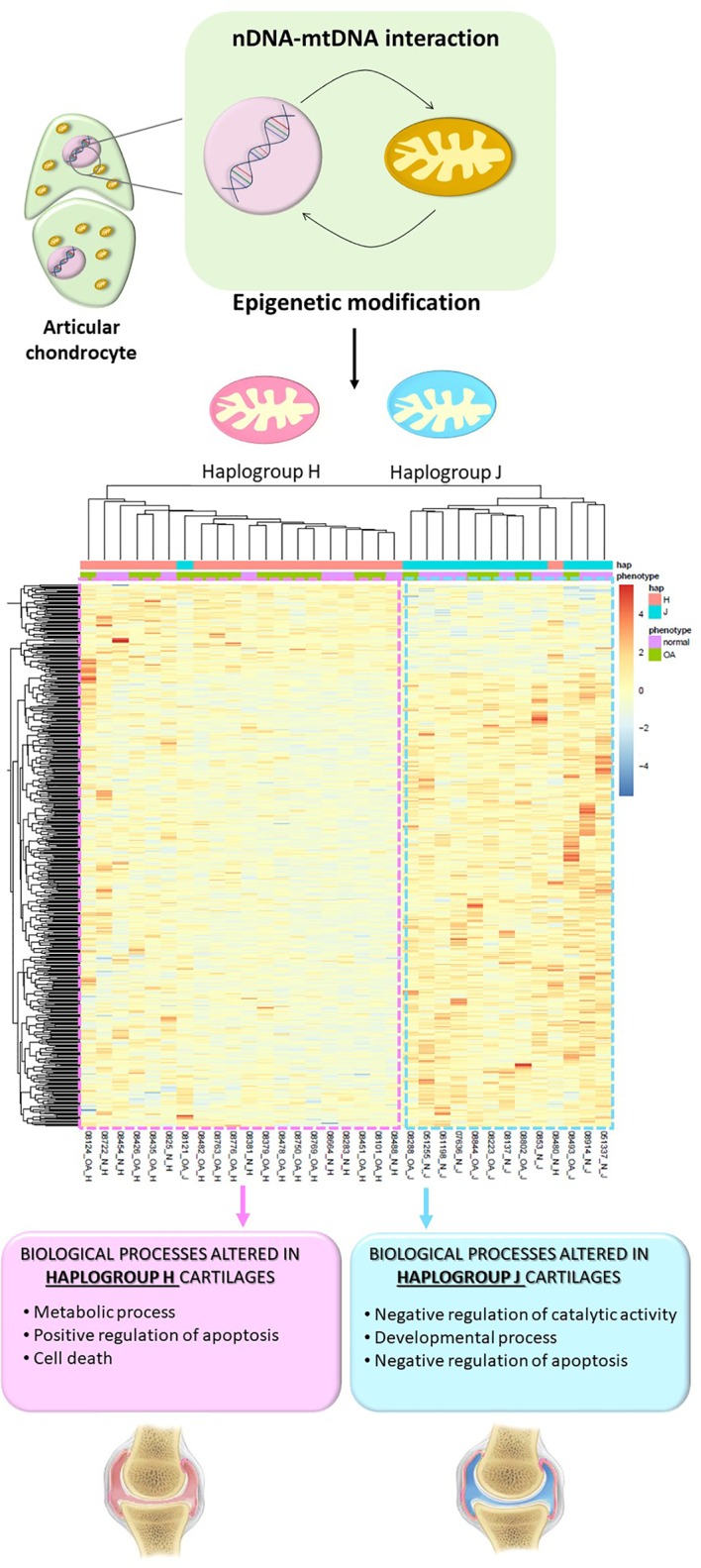
The interactions between the nucleus and mitochondria that take place inside articular chondrocytes give rise to epigenetic modifications that are mediated by mitochondrial DNA haplogroups. As a consequence, different haplogroup-associated methylation patterns condition key processes related to the development of OA. Permission is granted for publication of this figure as a modified version of the figure that appeared on page A17 of the July 2019 issue of Arthritis & Rheumatology (Clinical Connections).

In the case of OA, DNA methylation is involved in the phenotypic modulation that articular chondrocytes undergo during the OA process (Roach et al., 2005). After the few first studies, in which the methylation pattern of specific genes involved in OA pathogenesis was explored (Roach et al., 2005; Hashimoto et al., 2013), genome-wide DNA methylation assays were performed. These studies not only showed that knee and hip OA cartilages have different DNA methylation patterns but also identified a subgroup of OA patients with an enrichment of altered genes involved in inflammation and immunity (Fernandez-Tajes et al., 2014; Rushton et al., 2014). The only study that has so far analyzed the effect of mtDNA variation on the methylome of articular cartilage revealed that cartilages harboring haplogroups H and J show a differential methylation pattern, regardless of diagnosis. The study consisted of a genome-wide DNA methylation approach followed by a whole transcriptomic assay and demonstrated that apoptosis is enhanced in haplogroup H cartilage samples, together with an enrichment of overexpressed genes related to cell death; on the contrary, apoptosis appeared more repressed in haplogroup J cartilages. In addition, compared with H cartilages, samples with haplogroup J also showed a significant enrichment of hypomethylated CpGs of genes related to developmental process, including those belonging to the homeobox family of transcription factors, while H cartilages showed an enrichment of genes related to metabolic processes (Cortes-Pereira et al., 2019). These findings reflect that the epigenetic modifications that occur during the pathogenesis of OA and affect different key processes, such as metabolic alterations or apoptosis, vary depending on the mitochondrial genetic background, and this could determine the evolution of the disease (**Figure 1**).

Based on the description above, it is conceivable to consider the mitochondrial genome as an epigenetic regulator of the nuclear genome in articular cartilage. Stimuli such as reactive metabolic intermediates from the mitochondrial metabolism, small RNAs, mitochondria-derived peptides, and/or reactive oxygen species could be considered among the underlying mechanisms by which mitochondrial variation promotes modifications in the nuclear DNA methylome in OA (Schroeder et al., 2013; Horan and Cooper, 2014; Shadel and Horvath, 2015).

## Conclusions and Future Directions

The evidence presented in this review supports the hypothesis that mitochondrial genetics not only influences different features of OA disease but also modulates, in terms of mtDNA haplogroups, the nuclear DNA methylome of the only cell type present in articular cartilage, the chondrocyte. Based on this evidence, a broad field of promising research lies ahead.

Efforts must be made in the use of conplastic mice to investigate the influence of the mitochondrial background on specific OA-related features in animal models that develop spontaneous OA as well as in conplastic animals with induced OA. On the other hand, of special interest would be the design of transmitochondrial cybrids using chondrocytes as stable nuclear donors to subsequently test the influence of mtDNA variants in the native cells of articular cartilage. The use of both animal and cellular models, together with the methylation data originating from different genome-wide methylation studies, could contribute to the development of molecular biomarkers aimed at identifying specific OA phenotypes from the design of CpG classifier panels combined with the mitochondrial genetic background.

Given the role of adaptive selection in the origin of mtDNA haplogroups, and recognizing that they could be maladaptive in different environments with new lifestyles (Wallace, 2005), the proposed study of potential interactions between mtDNA variants and different nuclear DNA polymorphisms previously associated with OA susceptibility in various GWAS should be conducted taking into account the specific environment. Because none of the studies described in **Table 1** aimed to identify specific or single mitochondrial polymorphisms associated with different OA-related features, the precise identification through next-generation sequencing techniques of these specific mtDNA polymorphisms, characteristic or not of each haplogroup, from both isolated blood and articular cartilage from the same patient, would be a powerful tool for the consideration of mtDNA variation as a potential robust biomarker of OA.

In terms of therapeutic research, the restoration of mitochondrial function in OA chondrocytes would be the ultimate goal. This can be achieved by using different approaches to design different drugs that are capable of: i) suppressing mitochondrial oxidative damage and restoring extracellular matrix homeostasis (Farnaghi et al., 2017), ii) activating the AMPK-SIRT1-PGC1α pathway to induce mitochondrial biogenesis, therefore decreasing the pro-catabolic response of chondrocytes (Wang et al., 2015), iii) activating mitophagy, given its importance in preventing mitochondrial dysfunction (López de Figueroa et al., 2015), or iv) emulating the physiological effects of the OA-protective mtDNA haplogroup J on mitochondrial activity, as well as administering healthy isolated mitochondria into the osteoarthritic joint. On the other hand, given the bi-directional communication between the nucleus and mitochondria, interventions focused on the management of mitochondrial dysfunction by targeting the epigenome, or vice versa (Matilainen et al., 2017), would also be of interest.

## Author Contributions

FB and IR-P contributed equally to the design and coordination of the study; both conceived the study and participated in its design. AD-S and PR-L contributed to some of the findings described in the manuscript and helped to draft the final version of the manuscript.

## Funding

This work is supported by grants from Fondo de Investigación Sanitaria (CIBERCB06/01/0040-Spain, RETIC-RIER-RD16/0012/0002, PRB2-ISCIII-PT17/0019/0014, PI14/01254, PI16/02124 and PI17/00210) integrated in the National Plan for Scientific Program, Development and Technological Innovation 2013–2016 and funded by the ISCIII-General Subdirection of Assessment and Promotion of Research-European Regional Development Fund (FEDER) “A way of making Europe”. IR-P is supported by Contrato Miguel Servet-II Fondo de Investigación Sanitaria (CPII17/00026).

## Conflict of Interest

The authors declare that the research was conducted in the absence of any commercial or financial relationships that could be construed as a potential conflict of interest.
